# An agent-based model of triple-negative breast cancer: the interplay between chemokine receptor CCR5 expression, cancer stem cells, and hypoxia

**DOI:** 10.1186/s12918-017-0445-x

**Published:** 2017-07-11

**Authors:** Kerri-Ann Norton, Travis Wallace, Niranjan B. Pandey, Aleksander S. Popel

**Affiliations:** 1 Department of Biomedical Engineering, School of Medicine, Johns Hopkins University, Baltimore, MD 21205 USA; 2 Department of Oncology and the Sidney Kimmel Comprehensive Cancer Center, School of Medicine, Johns Hopkins University, Baltimore, USA

**Keywords:** Systems biology, Computational model, Breast cancer, Tumor heterogeneity

## Abstract

**Background:**

Triple-negative breast cancer lacks estrogen, progesterone, and HER2 receptors and is thus not possible to treat with targeted therapies for these receptors. Therefore, a greater understanding of triple-negative breast cancer is necessary for the treatment of this cancer type. In previous work from our laboratory, we found that chemokine ligand-receptor CCL5-CCR5 axis is important for the metastasis of human triple-negative breast cancer cell MDA-MB-231 to the lymph nodes and lungs, in a mouse xenograft model. We collected relevant experimental data from our and other laboratories for numbers of cancer stem cells, numbers of CCR5+ cells, and cell migration rates for different breast cancer cell lines and different experimental conditions.

**Results:**

Using these experimental data we developed an in silico agent-based model of triple-negative breast cancer that considers surface receptor CCR5-high and CCR5-low cells and breast cancer stem cells, to predict the tumor growth rate and spatio-temporal distribution of cells in primary tumors. We find that high cancer stem cell percentages greatly increase tumor growth. We find that anti-stem cell treatment decreases tumor growth but may not lead to dormancy unless all stem cells get eliminated. We further find that hypoxia increases overall tumor growth and treatment with a CCR5 inhibitor maraviroc slightly decreases overall tumor growth. We also characterize 3D shapes of solid and invasive tumors using several shape metrics.

**Conclusions:**

Breast cancer stem cells and CCR5+ cells affect the overall growth and morphology of breast tumors. In silico drug treatments demonstrate limited efficacy of incomplete inhibition of cancer stem cells after which tumor growth recurs, and CCR5 inhibition causes only a slight reduction in tumor growth.

**Electronic supplementary material:**

The online version of this article (doi:10.1186/s12918-017-0445-x) contains supplementary material, which is available to authorized users.

## Background

Breast cancer is a group of diseases that remain the most common malignancy afflicting women worldwide. Often targeted therapies focus on three main cellular receptors: estrogen, progesterone, and human epidermal growth factor receptor 2 (HER2) receptors. However, approximately 12% of all breast cancers lack these three targets [1]. Known as triple-negative breast cancer (TNBC) this subset is aggressive, metastatic, and difficult to treat. Lee and colleagues developed an accelerated metastasis xenograft model that increased spontaneous metastasis of TNBC primary tumor to the lungs [2]. They found that TNBC tumors secrete chemokines that increase metastasis to the lungs and from these experiments they identified several potential targets for triple-negative breast cancer [3–5]. Specifically, they found that there is crosstalk between the primary tumor and the lymphatic endothelial cells in the primary tumor site, the lymph nodes, and lungs. TNBC cells secrete interleukin 6 (IL6) that becomes systemic and ‘educates’ lymphatic cells at the primary and metastatic sites. The ‘educated’ lymphatic cells then increase production of C-C chemokine ligand 5 (CCL5) which increases pro-migratory signaling in the breast cancer cells through their CCR5 receptor. Other research has shown that mesenchymal stem cells secrete CCL5 promoting cancer cell motility, invasion, and metastasis [6]. Importantly, it has been shown that treatment with maraviroc, a CCR5 inhibitor approved by the FDA for HIV indication, reduced metastasis in lymph nodes and lungs [3, 7]. Maraviroc was also shown to reduce bone metastases from prostate cancer in animal models [8]. Since CCR5 is an important receptor for TNBC proliferation, migration, and metastasis, we wanted to understand the distribution of CCR5 in triple-negative cell lines and then use this information in computational modeling to predict how these factors affect tumor growth.

Cancer stem cells have been found in many types of cancer [9] including breast [10], colorectal [11], neuroblastoma [12], and lung [13]. They have been associated with therapeutic resistance in breast cancer [14] and may lead to recurrence [15]. The most common markers for breast cancer stem cells are CD44, CD24 and ALDH1 [16]. Specifically, breast cancer stem cells have been associated with TNBC [17]. In estrogen receptor positive MCF-7 cell lines, CCL5 could increase the number of cancer stem cells in the tumor [18]. Thus, we wanted to understand the contributions of breast cancer stem cells in triple-negative breast cancer cell lines.

Computational modeling is a useful tool for studying cancer [19] and predicting tumor response to therapies [20, 21], for reviews see [22, 23]; more specifically these methods can also be useful for understanding breast cancer, see review [24]. Early works investigating breast cancer using computational modeling include investigating the role of hypoxia and pressure in tumor necrosis [25], formation of pre-invasive tumor architectures [26], conditions leading to the disruption of normal breast acini formation [27, 28], patient calibrated breast cancer prediction [29], and predicting drug response [30]. More recently, computational models have been used to study ductal carcinoma in situ (DCIS) progression into invasive cancer [31], mechanical stress in breast cancer [32], interactions with the tumor microenvironment [33], and calcifications in the breast [34] . We have used agent-based models to investigate DCIS progression [35], avascular tumor growth [36], and tumor angiogenesis [37]. We now extend these approaches to understand the role of receptor heterogeneity in triple-negative breast cancer.

Cancer stem cells have also been the topic of a large number of computational models [38]. A series of models by Enderling and colleagues have investigated cancer stem cell dynamics in relation to radiotherapy resistance [39], directed migration [40], immunity [41], tumor dormancy [42], and senescence [43]. One interesting finding was that cell death could actually accelerate tumor growth due to the fact that it left space for stem cells to proliferate [44]. In particular, the inclusion of cancer stem cells in models were demonstrated to more accurately represent invasive behavior than those that only included non-stem cells [45]. Michor and colleagues have used computational models to study stem cell dynamics in pancreatic cancer [46], glioblastoma [47], and colorectal cancer [48]. Others have focused on cancer stem cells’ ability to evade the immune system [49]. In the present study, we measure the fraction of cancer stem cells in a TNBC cell line and also assemble relevant data from other researchers; we then examine how these numbers affect tumor growth using computational modeling.

Tumor heterogeneity is an important aspect of breast cancer growth and has been the subject of many reviews [50–52]. Breast cancer heterogeneity is considered a major contributor to the difficulty in eradicating the disease including drug resistance [53–55]. In this paper, we examine receptor heterogeneity in triple-negative human breast cancer cells lines. The numbers of CCR5 cell surface receptors and molecular markers governing stemness were assembled from our and other laboratories. We then build an agent-based (rule-based) model of tumor growth for triple-negative breast cancer cells. We compare the growth rates and tumor morphology under different conditions, such as different stem cell and CCR5+ cell fractions, as well as drug treatment and hypoxia.

## Methods

### In silico agent-based model

**Fig. 1:**
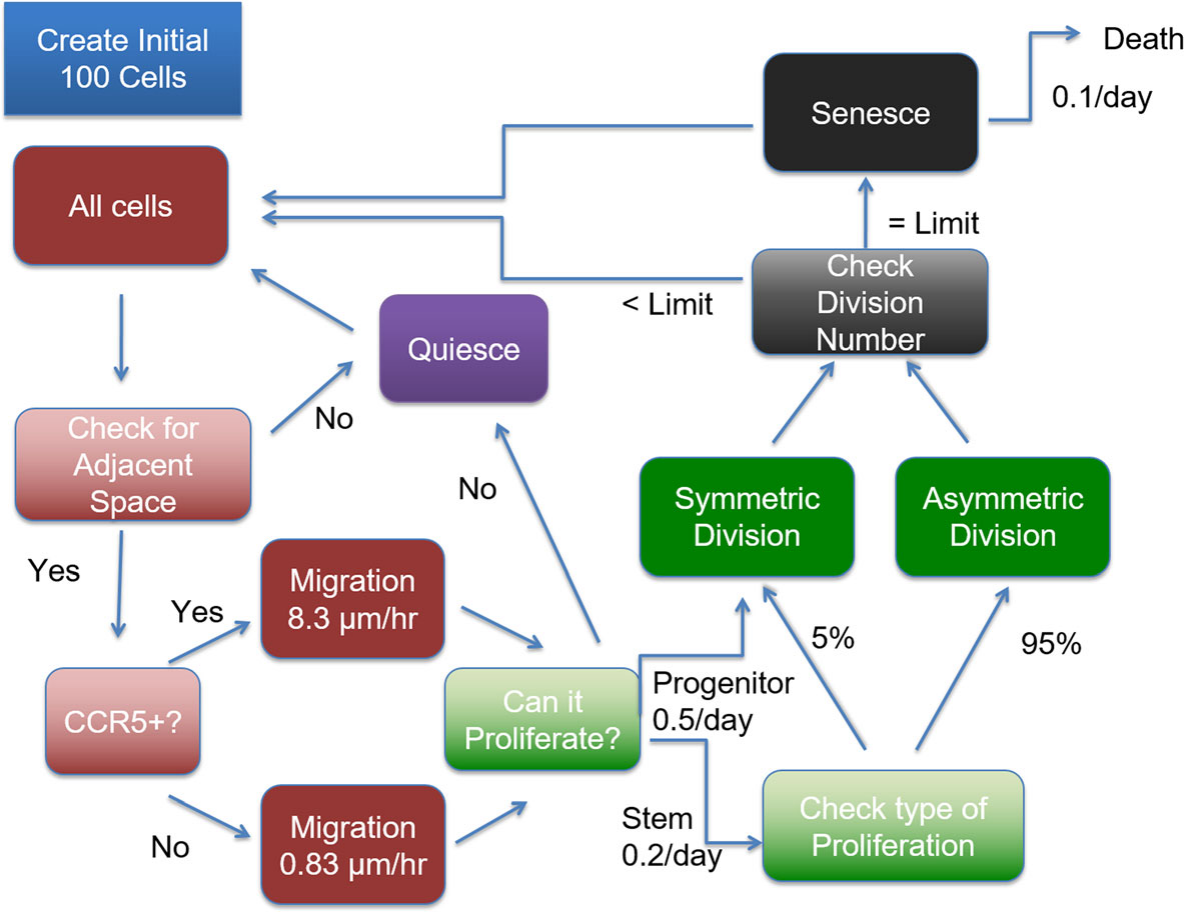
Flowchart of the Agent-Based Model. The initial 100 cells are set up based on the flow cytometry results for the cell line MDA-MB-231 (MB231). These initial 100 cells were placed in a cubic grid 100x100x80 voxels, each voxel 20x20x20 microns, one cell per voxel. The cells become quiescent if they have no adjacent space. The cells migrate based on their CCR5 expression levels. Stem cells can divide symmetrically or asymmetrically, whereas progenitor cells divide only symmetrically. If a progenitor cell has reached its division limit, it becomes senescent. Each day a senescent cell has a 10% chance of dying

The flow chart of the model is shown in Fig. 1. We initiated each simulation with 100 triple-negative breast cancer cells; the distributions of CCR5+ and stem cells matched those found in flow cytometry in triple-negative breast cancer cell lines. The initial 100 cells were placed in a cubic grid 100x100x80 voxels, each voxel 20x20x20 μm, maximum of one cell per voxel. The MDA-MB-231 cell diameter has been measured to be approximately 20 microns [56]. We assemble the data from the literature on percentages of CCR5+ cells in several breast cancer cell lines; they range from 1% to 14% under different treatments, including our data presented in the Additional file 1, Table 1. We have also found the percentages of cancer stems cells in different breast cancer cell lines range from less than 1% to 34%, including our data presented in the Additional file 1, Table 2. All simulations were run for at least 8 different times and averaged.

**Table 1:** Percentages of CCR5+ Cells

CCR5+ percent	Cell Line	Conditions	Reference
6.9%	Basal	CCR5 numbers	[7]
9%	MDA-MB-231	CCL5 responsive cells	"
3%	MDA-MB-231 + maraviroc	"	"
11%	MDA-MB-231	"	"
1%	MDA-MB-231 + vicriviroc	"	"
13%	HS578T	"	"
5%	HS578T + maraviroc	"	"
14%	HS578T	"	"
5%	HS578T + vicriviroc	"	"
5–7%	SUM149	CCR5 numbers	[82]
2–6%	MDA-MB-231	CCR5 numbers	Our data

**Table 2:** Percentages of cancer stem cells

Stem cell %	Cell line	Conditions	Reference
20%	SUM147	control	[83]
10%	SUM147	treatment	[83]
1–15%	MDA-MB-231		[84]
1%	MDA-MB-231	ALDH1	[85]
12%	MDA-MB-231	Paclitaxel	"
1–13%	SUM159	with/− Paclitaxel	"
6–24%	SUM149	"	[86]
8–34%	SUM159	"	"
0.2%–12%	tumors	ALDH1	[87]
3.10%	MDA-MB-231		[88]
2.70%	MDA-MB-435		"
~0.5%	SUM225	CD44^+^/CD24^−^/ESA^+^	[89]
~2.5%	SUM149	CD44^+^/CD24^−^/ESA^+^	"
~1.75%	SUM159	CD44^+^/CD24^−^/ESA^+^	"
~2.5%	SUM1315	CD44^+^/CD24^−^/ESA^+^	"
~2%	MDA-MB-231	CD44^+^/CD24^−^/ESA^+^	"
9–20%	MDA-MB-231	CD44^+^/CD24	Our data

In the initial simulations with MDA-MB-231 (in some instances referred to as MB231 for brevity) tumors, we used CCR5+ percentages as 6% and cancer stem cell percentages as 20% based on our experimental results. We then vary these numbers in the sensitivity analysis. For MB231 cells, we placed nineteen CCR5- stem cells, one CCR5+ stem cell, five CCR5+ progenitor cells, and seventy-five CCR5- progenitor cells on the grid. Then the simulation ran through all the cells randomly. First, the neighboring positions (each voxel has 26 neighbors along x,y,z axes and diagonally, also known as the Moore neighborhood) of each cell were checked to determine whether there was a free space, if not the cell became quiescent. Each cell has a CCR5 receptor level that determines its migration speed. If a cell was CCR5+, it was set to migrate faster than a CCR5- cell. Then it was determined whether the cell could proliferate; if the cell was a progenitor cell it must divide symmetrically into two progenitor cells, otherwise the stem cell could divide symmetrically into two stem cells or asymmetrically into a stem cell and a progenitor cell. If a progenitor cell had reached its division limit the cell became senescent. With each iteration a senescent cell had a 10% chance of dying. Once a cell dies, the voxel it occupied becomes available for other cells to proliferate or migrate into. The simulations run for 1080 days or until the tumor reaches 500,000 cells.

### Quiescence

We randomly selected each cell and checked whether all of its neighboring spaces were occupied. If so, the cell became quiescent and was no longer able to proliferate or migrate. If one of the neighboring spaces became unoccupied the cell reverted back to its proliferative state.

### Migration

In this model migration was governed by the cells’ CCR5 status and its microenvironment. Migration occurred as a random walk, where a neighboring space (Moore neighborhood) that was unoccupied was selected randomly and the cell was moved to that location. Based on reported observations of breast cancer cell migration rates, Table 3, we assumed that as reference values, if a cell was CCR5-, it could move one cell length per iteration (0.83 μm per hour or 20 μm per 24 h equivalent to one iteration), the lower end of the migration rates from in vitro studies [36, 57, 58]. CCR5+ cells ranged from moving 2 cell lengths per iteration (1.67 μm per hour) to 20 cell lengths per iteration (16.67 μm per hour), based on the intermediate migration rates from in vitro studies [7, 59–61]. The high values were used for the more migratory hypoxic cells. If a MB231 cell was in a hypoxic microenvironment, it became more migratory [62]. Therefore in the model, a cell in a hypoxic zone could move three times as fast as a non-hypoxic cell each time it migrated [62].

**Table 3:** MB231 migration rates

MB231 Migration rate	Substrate	Conditions	Reference
0–40 μ/hr	GelMA hydrogels	2D	[57]
0.1–4 μ/hr	Matrigel	3D	[58]
12–48 μ/hr	Collagen	2D	[60]
8–18 μ/hr	-	channel	[59]
38 μ/hr	Fibronectin	2D	[90]
7 μ/hr	Collagen	3D	[91]
3–9 μ/hr	Collagen	3D	[92]

### Proliferation

Proliferation was governed by the cell’s state, by whether it was a stem or a progenitor cell, and by its microenvironment. A stem cell could proliferate symmetrically to produce two stem cells or asymmetrically to produce a stem cell and a progenitor cell. A progenitor cell could only proliferate symmetrically to produce two progenitor cells. A stem cell had a range between 10% and 40% probability to proliferate each day and a symmetric division rate of 5% as done in previous models [36]. We assumed that as reference values a stem cell had a 20% probability of proliferating each day and a progenitor cell had a 50% probability of proliferating each day under normoxic conditions (36). Under hypoxic conditions the rates of proliferation were cut in half [62].

A cell could only proliferate if it had free adjacent space. Thus, the cell checked whether any spaces within the surrounding 26 grid spaces were unoccupied and chose a random free space to be the position of the new cell. A progenitor cell also checked whether it had reached its division limit before proliferating. If it had, it became senescent. A stem cell or progenitor cell had a 5% chance of producing a new CCR5+ cell each time it symmetrically proliferates. Once the cell had proliferated, its cycle number was increased by one and the new cell was assigned the same cycle number as its parent. The new cell was then placed on the grid.

### Senescence

Stem cells could proliferate for the entire span of the simulation whereas progenitor cells could only proliferate 12 times before they became senescent, as done in previous models [36]. Once they were senescent they could no longer proliferate. Each day a senescent cell had a 10% probability of dying. Once a cell dies it is removed from the simulation.

### In silico anti-stem cell treatment

Several drugs have been reported to selectively target breast cancer stem cells, such as salinomycin [10]. We assumed in the model that an anti-cancer stem cell drug would kill a percentage of cancer stem cells. We administer the drug *in silico* after day 150. We apply the drug, with specific stem cell death rates between 50% to 90% efficacy, every 2 weeks for 98 days. We then track the growth of the tumor after treatment.

### In silico maraviroc treatment

Maraviroc is an FDA approved drug for HIV, a C-C chemokine receptor 5 (CCR5) inhibitor [63]. It has been shown to reduce metastasis to the lungs in a MB231 xenograft model in nude mice [3]. We assumed that maraviroc was able to reach all cells and was effective at shutting down the CCR5 enhanced migration. The therapeutic was applied throughout the entire simulation, which is not entirely realistic but would represent the most ideal case. Therefore, CCR5+ cells were considered inhibited and behaved as CCR5- cells, such that they could only move 0.83 μm or one cell length every day in the presence of maraviroc.

### In silico hypoxia

We assumed the vasculature was placed along the y-axis, assuming that this is the location of the normal tissue and the tissue was well oxygenated. Any cell greater than a distance of 200 μm away from the vessels became hypoxic [64]. Experimentally, proliferation assays have shown that in vitro MB231 cells are half as proliferative under hypoxic conditions [62]. Migration assays have shown that in vitro hypoxic MB231 cells were also around 3 times as migratory as normoxic cells [62]. Thus, a cell would migrate three times as much in a hypoxic region than in a normoxic one. Specifically, a cell would search for an open space and move into this space and then it would search for a new open space and move. This random search would happen three times and is prevented from moving back to its original position. The numbers of CCR5+ cells increase under hypoxia [62] and we modeled the number of CCR5+ cells from MB231 cells as 25% based on the ranges from the literature [62]. Thus, a progenitor cell in a hypoxic region would produce a CCR5+ cell 25% of the time it proliferated, on average. We perform simulations to see how hypoxia affects the overall growth of the tumors.

### Model implementation

The model proceeds in a stepwise fashion, in which the decisions made each day are based on the conditions and environment of the previous day. Each cell is confined to a single grid space (voxel) in a cellular automata (on-lattice) system and each cell is an automatous agent that makes decisions and performs actions based on its intrinsic parameters and its microenvironment in an agent-based system. The external boundary conditions of the grid are static, such that no cell can leave the grid. Once a cell hits a boundary it can only move in a direction within the grid space. The grid size is 4x4x4 mm with each cell having a diameter of 20 μm. The default number of initial stem CCR5-, stem CCR5+, progenitor CCR5+, and progenitor CCR5- cells are 19, 1, 6, and 74 respectively. The model was implemented in Matlab (MathWorks, Natick, MA).

### Shape metrics

We calculated several metrics related to the 3D shape of the tumors: chord ratio, a variation of circularity [53], chord length [54], moment of inertia [53, 54], and fractal dimension [55, 65–67]. The chord ratio metric is calculated by the average d_c_/r_s,_ where r_s_ is the radius of a sphere with the same volume as the tumor and d_c_ is the Euclidean distance from each of the cells on the periphery of the tumor and the tumor centroid. The chord length is the average radial distance/ the maximum radial distance, average d_c_/max(d_c_). The moment of inertia is defined as

$$ \sqrt[]{\left[\frac{1}{N}\sum_{i=1}^N{\left({z}_i-\mu \right)}^2\right]} $$

where z_i_ are values of the radial distance function, N are the cells on the perimeter of the tumor, and μ is the mean radial distance. The moment of inertia is normalized by the mean radial distance. For both the chord ratio and moment of inertia, the larger the number the more finger-like the morphology. For the chord length, the smaller the number the more finger-like the morphology.

## Results

### In silico CCR5+ and stem cell percentage parameter space

**Fig. 2:**
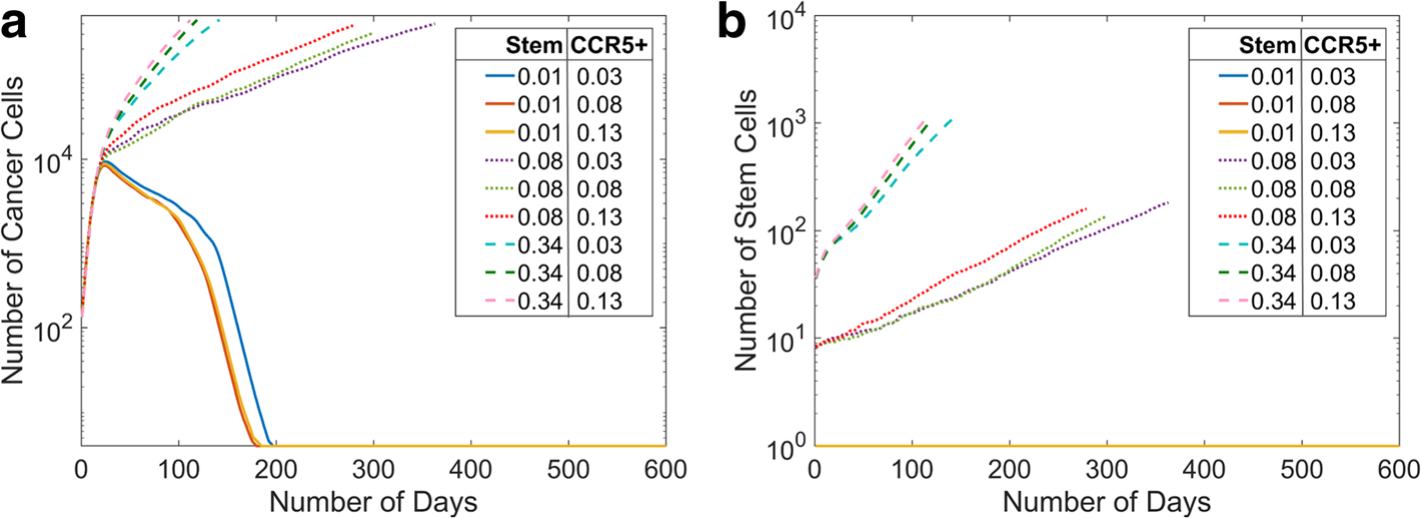
Changes in the Initial Percentages of Cancer Stem Cells and CCR5+ Cells. We varied the initial cancer stem cell percentages from 1% to 34% and the initial CCR5+ percentages from 3% to 13%. We show the mean cell data over time (**a**), and the mean stem data over time (**b**). The percentages of cancer stem cells have a greater effect than the differences in CCR5+ cells

Triple-negative breast cancer cell lines were found to have varying CCR5+ percentages between 1 and 15% (Table 1) and varying cancer stem cell percentages between 0 and 34% (Table 2). Therefore, to reflect the different experimental measurements and explore the entire ranges of these parameters reported by different researchers, we varied CCR5+ percentages between 3 and 13% and the stem cell percentages *in silico* between 1 and 34%. We find that at very low stem cell numbers the tumors initially grow but then shrink until there is just one stem cell left, Fig. 2. The percentage of stem cells has more effect on the total population but for intermediate percentages of cancer stem cells the CCR5+ percentage has more of an effect on the tumor growth, Fig. 2.

**Fig. 3:**
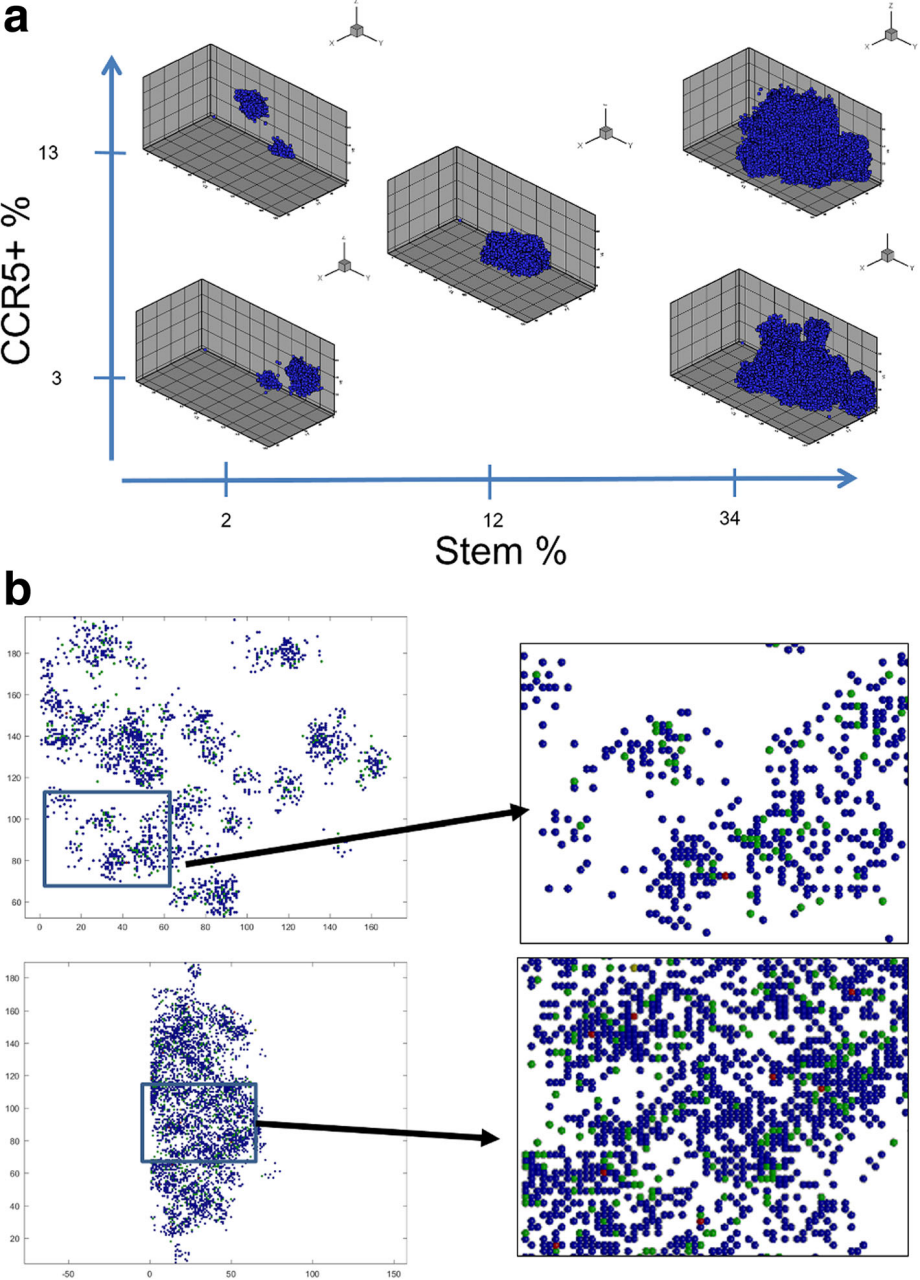
3D Simulation Plots of the MB231 with Different Stem Cell and CCR5+ Populations. **a** Lower stem cell populations and higher CCR5+ percentages lead to more fingering morphologies. The plots are at day 100. **b** The top tumor is a cross section of a tumor for simulation with 2% stem cells and 13% CCR5+ cells, with an inset. The bottom tumor is a cross section of a tumor for simulation with 34% stem cells and 13% CCR5+ cells, with an inset. With lower stem cell initial percentages, the tumor is made up of clusters of cells whereas the higher stem cell percentages result in a single larger tumor. Progenitor cells are shown in *blue*, CCR5+ cells in green, stem cells in *red* and both CCR5+ and stem cells in *yellow*

We show the parameter space of tumors at day 100 with varying cancer stem cell and CCR5+ percentages, Fig. 3a. The CCR5+ percentages range between 3% and 13%. We show the stem cell percentages between 2% and 34% because for 1% there is only one cell. At high stem cell percentages, the tumors grow very quickly. The higher CCR5+ percentage with high stem cell percentages also has a more compact shape than the lower CCR5+ percentage. At lower cancer stem cell percentages, the tumors grow much slower and are more spread out. At intermediate values of CCR5+ and cancer stem cell percentages the tumor is of a medium size and has a compact morphology.

### At high CCR5+ percentages, the cancer stem cell percent governs the in silico morphology

To examine the distributions of stem cells, CCR5+ cells, and progenitor cells we analyze slices from the 3D tumors. We show two examples with a high percentage of CCR5+ cells (13%) and a low (2%) initial percentage of stem cells, Fig. 3b, compared to a high initial percentage of stem cells (34%), Fig. 3b. While CCR5+ cells (green cells) are about the same, the number of stem cells (red cells) are much smaller. Clearly the high percentage of initial stem cells results in a more compact tumor, whereas a low percentage of initial stem cells results in a disperse tumor made of several tumor “self-metastases.” Thus, while having smaller stem cell percentages results in slower growing tumors, the tumors that result may be harder to excise completely.

### In silico anti-stem cell treatment decreases tumor size for MDA-MB-231 cells

**Fig. 4:**
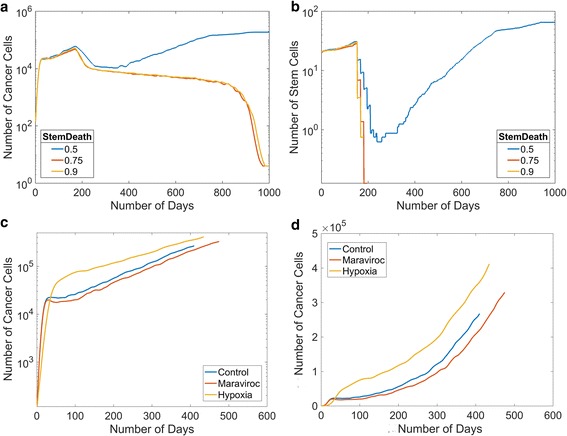
Simulated tumor growth of MB231 cells under different conditions. **a** We investigate three doses of a drug treatment targeting cancer stem cells starting at day 150 with varying efficacy at killing cancer stem cells between 50%–90%. Plot of the mean total cell numbers over time after multiple doses of treatment. **b** Plot of the mean stem cell numbers over time after multiple doses of a drug treatment targeting cancer stem cells. **c** A semi-log plot comparison of MB231 primary tumors under control, hypoxic, and maraviroc conditions. **d** A plot of MB231 primary tumor under control, hypoxic, and maraviroc conditions

The previous simulations have examined the ranges of CCR5+ and cancer stem cells, for examining response to therapeutics we modeled MB231 cells with 6% CCR5+ cells and 20% stem cells based on our and others experimental results, see Tables 1 and 2. Several drugs are reported to selectively target breast cancer stem cells, such as salinomycin [10]. We treated MB231 tumors in silico starting at day 150 with multiple doses of a drug that selectively targets and kills stem cells and assumed that it killed between 50%–90% of cancer stem cells, Fig. 4a,b. We investigate multiple doses of the same stem cell killing drug starting at day 150 and administering it every two weeks until day 248. Under multiple doses with 75% or 90% efficacy the stem cells eventually die out and the tumors slowly die over 750 days, Fig. 4a,b. Once all the progenitor cells have reached their division limit they quickly die off. With only 50% efficacy, in some cases all the stem cells die off and in others some remain. If any stem cells remain, the stem cells eventually grow in numbers past the number they were at when they were originally killed off, Fig. 4b. In all three conditions, the tumors survive for hundreds of days even though they are dying off, Fig. 4a, thus even effective stem cell treatments will not quickly eliminate the tumors. We find that one dose killing 50–90% of stem cells merely slows down the growth of the tumor, data not shown. In one particular simulation, eliminating 90% of cancer stem cells caused the number of stem cells to drop to one and the tumor to remain dormant for some time but the number of stem cells eventually starts increasing again, data not shown. These results indicate that completely eliminating stem cells is necessary to eventually kill off the tumor and even if the treatment is effective at eliminating all stem cells, the regression of the tumor may be very slow.

### In silico maraviroc treatment slightly decreases tumor growth for MDA-MB-231 cells

Maraviroc is an FDA-approved CCR5 inhibitor. We modeled maraviroc treatment by decreasing the migration rate of CCR5+ cells to be the same as CCR5- cells. Since the growth curves of the CCR5+ cells are more migratory, we hypothesized that maraviroc treatment might decrease the overall growth of the tumor due to an escape from spatial inhibition. We performed simulations of MB231 cells, Fig. 4c,d, under control and in silico maraviroc treatment conditions. We found that maraviroc treatment slows the growth of the MB231 tumors. We find that at day 300, which occurs after the initial growth phase but before the simulations have ended, the maraviroc treated MB231 tumors had fewer cells than control. The simulations also show that the day the tumor reaches 500,000 cells is somewhat higher for maraviroc treated tumors than control, see below. All the tumors exhibit an exponential growth curve with an R^2^ value of 0.99.

### Hypoxia increases tumor growth for MDA-MB-231 tumors

We included the development of hypoxic regions within the tumor and simulated the growth of MB231 tumors, Fig. 4c,d. The growth of MB231 tumors is faster under hypoxic conditions. We also find that at day 300, the hypoxic MB231 tumors had significantly more cells than control. The simulations also show that the length of time for the tumor to reach 500,000 cells is not significantly different for hypoxic tumors than for control tumors, see below.

### In silico MDA-MB-231 tumors have solid and invasive 3D morphologies

**Fig. 5:**
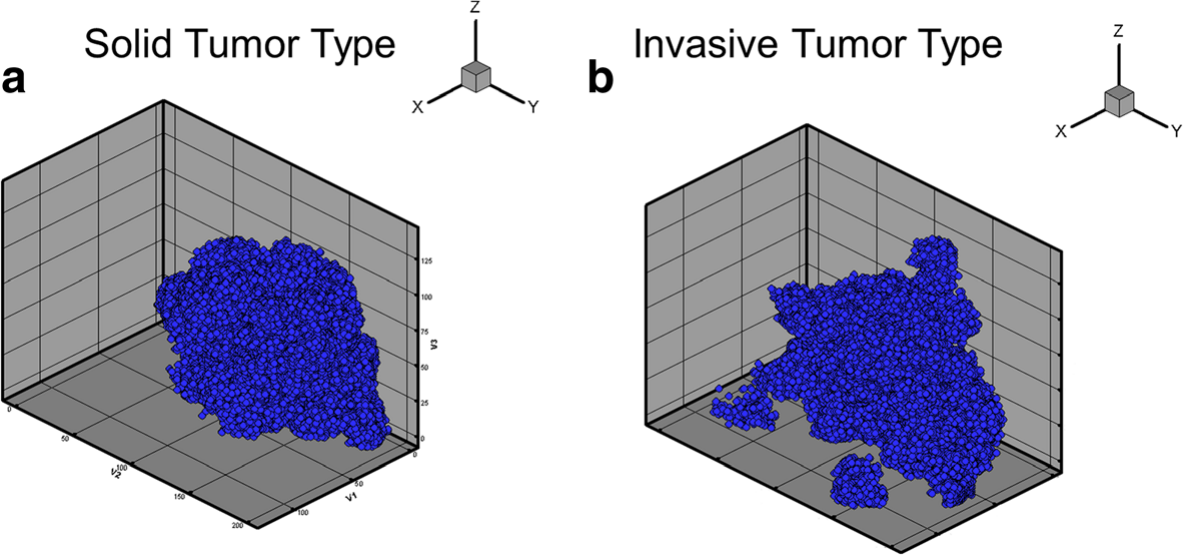
3D Simulation Plots of the MB231 Solid and Invasive Morphologies. **a** An example of an MB231 tumor with a solid morphology. **b** An example of a tumor with an invasive morphology. There are finger-like projections coming out of the tumor making it a more invasive morphology

We find that even though the same initial parameters are used to simulate MB231 tumor growth, their ultimate morphologies vary between a solid, more compact tumor and an invasive, fingering tumor. An example 3D image of a solid MB231 morphology is shown in Fig. 5a. This tumor has a chord ratio metric of 1.18 and a moment of inertia of 0.23. In contrast, the MB231 tumors shown in Fig. 5b have finger-like projections, which is characteristic of invasive tumors. These fingers are due to isolated stem cells that are surrounded by progenitor cells which form due to migration. This tumor has a chord ratio metric of 1.36 and moment of inertia 0.35. We evaluate the differences in metrics under different conditions below.

### Stem cell proliferation rate has a greater effect on tumor growth than migration rate

**Fig. 6:**
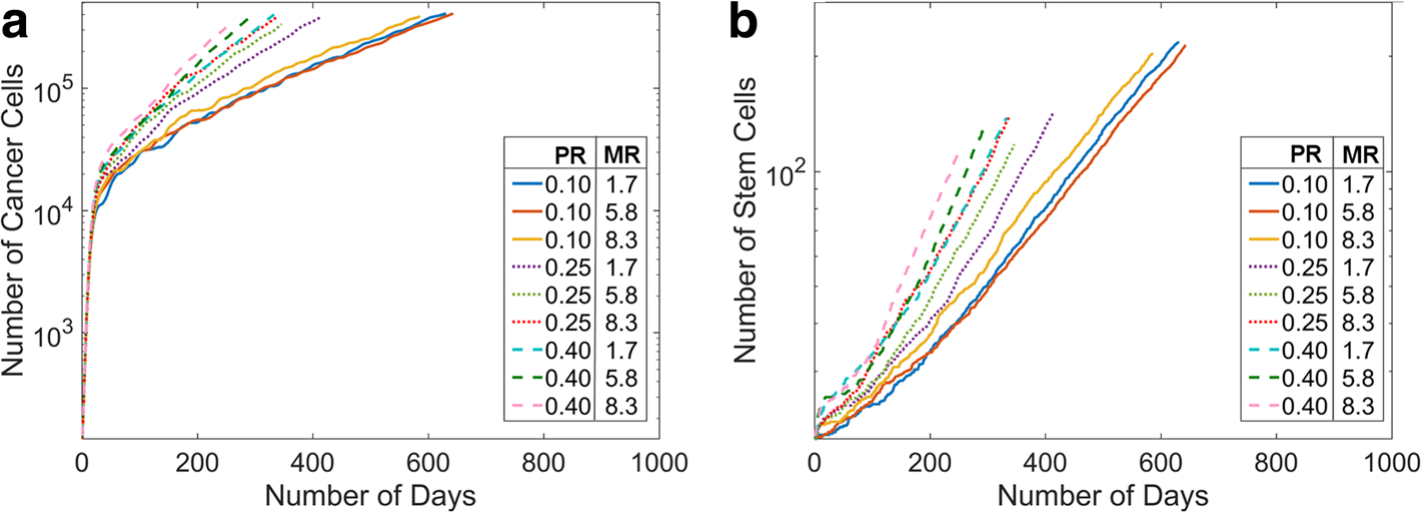
Cell Growth under Different Stem Cell Proliferation and CCR5+ Migration Rates. **a** MB231 total cell growth under different proliferation and migration rates (PR, 1/day; MR, μ/h). Lower stem cell proliferation rates lead to slower growth. **b** Mean stem cell number under different proliferation and migration rates. Lower stem cell proliferation rates leads to fewer stem cells over time

In order to determine the effects of stem cell proliferation and CCR5+ cell migration on the overall tumor growth, we performed simulations varying the stem cell proliferation rate between 0.1 and 0.4 per day and the CCR5+ migration rate between 0.8 and 5.8 μm per hour, in accordance with experimental data, see Tables 1-3. The mean cell numbers over time for each parameter condition are shown in Fig. 6a, and the mean numbers of stem cells over time in Fig. 6b. We do not show mean CCR5+ data over time because they follow the same trends as mean cell data. Low proliferation rates (0.1 per day) (PR) were associated with much slower tumor growth than higher proliferation rates. In contrast, changes in migration rates (MR) had less effect on tumor growth. We find similar trends for mean stem cell growth over time, Fig. 6b. Low proliferation rates slow stem cell growth over time and variation in migration rates have little effect.

### 3D morphology parameter space

**Fig. 7:**
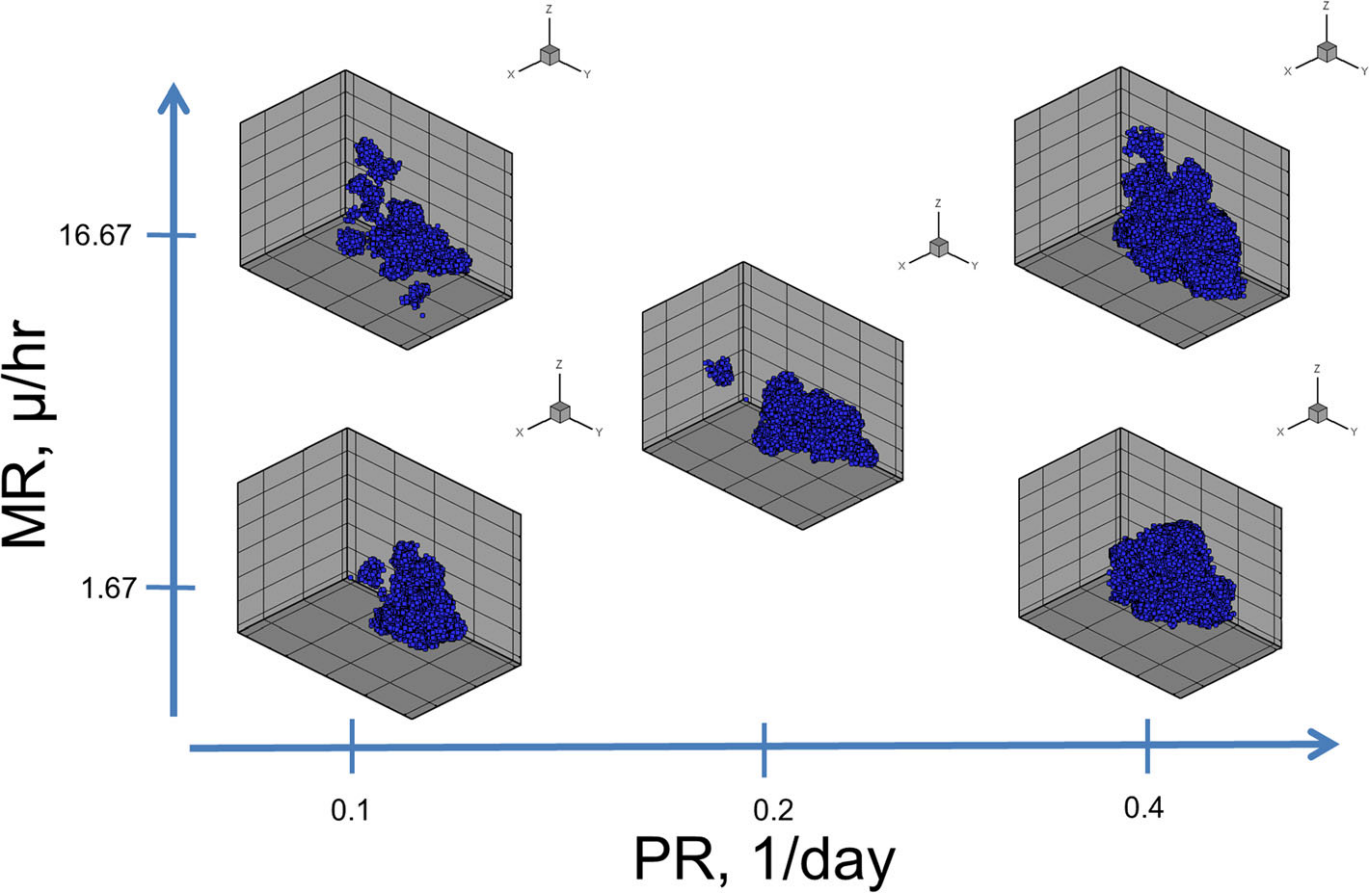
3D Simulation Plots of the MB231 Tumors under Different Migration and Proliferation Rates. Lower proliferation rates (PR) and higher migration rates (MR) lead to more fingering morphologies

We display the 3D morphologies of the tumors under different proliferation rates (PR) and migration rates (MR) at 300 days, Fig. 7. Migration appears to have more influence on the morphology and proliferation affects the numbers of cells. Tumors with higher MR have more finger-like projections, invasive bumps growing off of the tumor, than tumors with lower MR rates. Higher PR increases the numbers of cells and also contributes to a more solid-like morphology. The most finger-like tumor had low PR and high MR. The solid type morphologies are predicted to be less invasive, whereas the finger-like projections are predicted to be more invasive since they resemble invasive fronts seen in cancers.

**Fig. 8:**
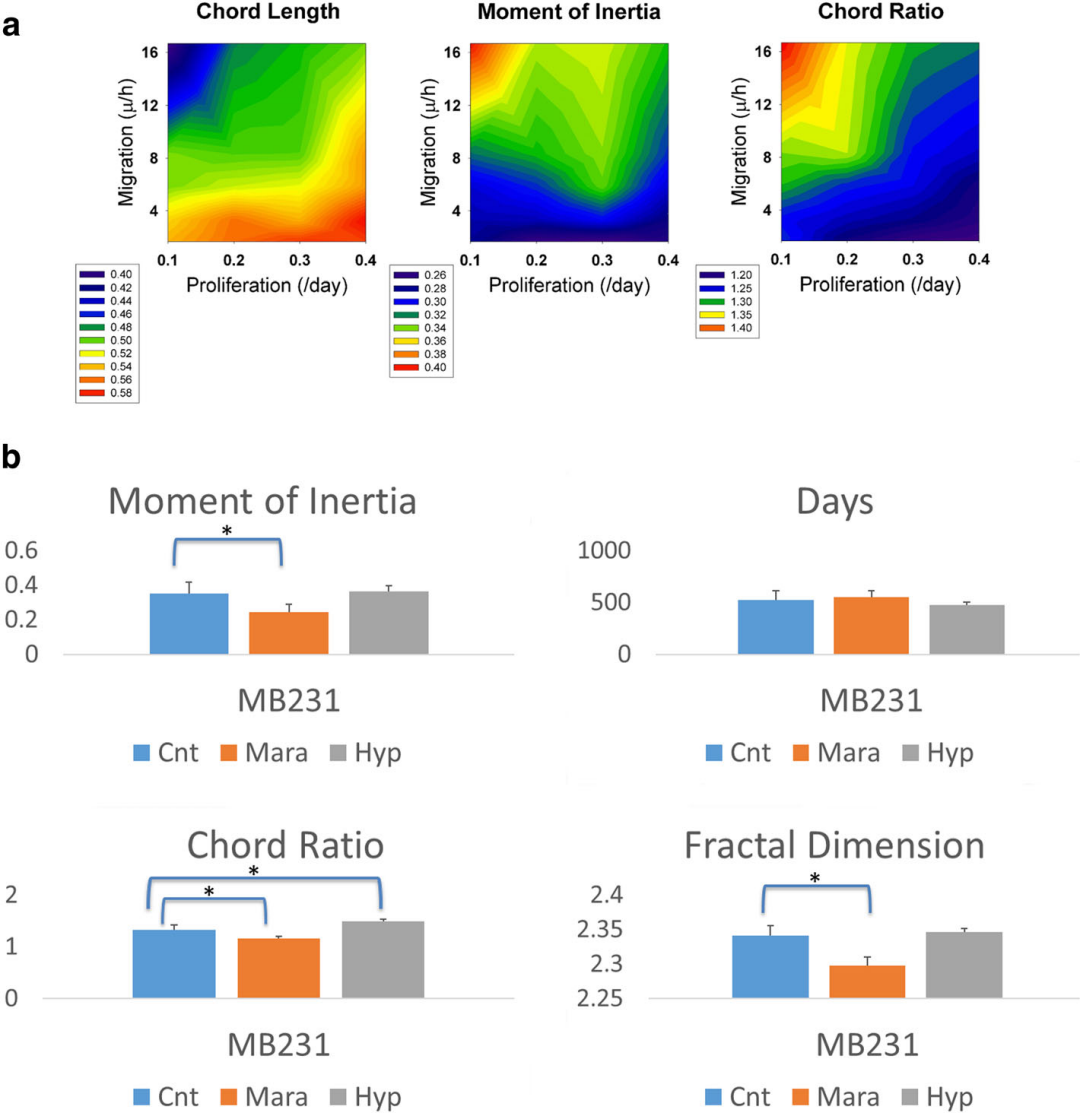
Comparison of Shape Metrics of Tumors. **a** Contour plot of the chord length with increasing migration and proliferation rates. Contour plot of the moment of inertia with increasing migration and proliferation rates. Contour plot of the chord ratio metric with increasing migration and proliferation rates. All metrics show that the fingering morphology increases with increasing migration and decreasing proliferation. **b** The shape metrics indicates the invasiveness of the tumor morphology. We evaluate moment of inertia, days until end of simulation (500,000 cells), chord metric and fractal dimension. MB231 cells under maraviroc (Mara) treatment have statistically significant lower shape metric values than the parent cell lines (Cnt). MB231 cells under hypoxic conditions (Hyp) have statistically significant higher chord metrics than the parent cell lines (Cnt). Simulated maraviroc treated tumors all have statistically significant less invasive morphologies than their parent lines

To get a quantitative assessment of the effects of proliferation and migration we evaluated three shape metrics (chord length, moment of inertia, and chord ratio), and we display the metrics as contour plots for a range of PR and MR values, Fig. 8a. The lower the value of the chord length, the more finger-like the morphology. The higher the value of the chord ratio and moment of inertia the less compact the tumor shape is and thus they have more fingers and/or more prominent fingers. From these contour plots, it is clear that the most finger-like morphology is at high levels of migration and low levels of proliferation, whereas the tumor is more spherical at low migration and high proliferation rates.

### Contribution of maraviroc treatment and hypoxia to the invasive morphology of the tumor

We examined four metrics: moment of inertia, day at which the tumor reaches 500,000 cells, chord ratio, and fractal dimension for MB231 tumors under control, maraviroc, and hypoxic conditions, Fig. 8b. We found that for the maraviroc treated tumors all metrics were statistically lower than for the normal condition for all metrics except for days using a one-tailed t-test, indicating a more compact morphology. We found that chord ratio was statistically higher for hypoxic than control conditions using a two-tailed t-test, indicating a more finger-like morphology. Also, hypoxic treated tumors took fewer days to reach 500,000 cells than the control tumors.

## Discussion

High numbers of CCR5 receptors allow for an increased migratory behavior in tumors. We predicted that the increased migration might also increase tumor growth by opening up new space into which cells may divide. We found that the tumor growth was slightly slower by simulated maraviroc treatment, which was assumed to reduce the CCR5+ migration rate to normal. This result is qualitatively consistent with the studies from our laboratory showing that primary tumor growth was slightly lowered by maraviroc monotherapy treatment (but significantly inhibited by maraviroc in combination with other agents) (K. Jin, unpublished data) and observations by Lee et al. showing that the primary tumor was unchanged but the incidence of lung metastases was decreased [3]. We find that at lower migration rates, the decreased tumor growth does not occur (data not shown).

Stem cell proliferation and CCR5+ migration rates were found to influence the tumor growth and morphology. However, despite the importance of both these attributes, the effects of proliferation were more significant. Lower initial cancer stem cell percentages can greatly lower the growth of the tumor. Lower stem cell proliferation rates decreased the total number of tumor cells, the number of CCR5+ cells, and the number of stem cells. Lower stem cell proliferation and higher CCR5+ migration rates were correlated with having a more finger-like morphology. This was observed qualitatively with the 3D morphologies and verified by the shape metrics. Each stem cell generates a group of progenitor cells that surround it. Therefore, if there are large amounts of stem cells these progenitor cell groups will overlap with one another, which would lead to a more solid-like morphology. When there are fewer stem cells these progenitor cells can form finger-like projections as they move away from the main tumor body. Thus, a lower stem cell proliferation rate can lead to a more finger-like morphology. The more migratory the cells, the more they can form finger-like projections as well due to a cell leaving a “trail” of cells behind them as they migrate. This is consistent with other models that showed that having fewer stem cells leads to a more invasive morphology [40, 45].

This computational model focuses on heterogeneity, specifically two relevant types of breast cancer heterogeneity are distributions of cancer stem cells and chemokine cell surface receptors [68, 69]. We find that the distribution and number of stem cells greatly influences the morphology of the tumor, with lower numbers of stem cells leading to clusters of cells surrounding stem cells similar to ‘self-metastases’ [40, 42]. We find that the higher percentages of CCR5+ cells lead to more stem cell clusters due to the fact that migration leaves space for stem cells to migrate and proliferate. The heterogeneity of stem cells and CCR5+ cells leads to differences in the growth and morphology of the tumor. In other computational models, the migration rate of cells and cell turnover were found to contribute to the increased tumor heterogeneity [70].

We model a targeted cancer stem cell therapy, such as salinomycin [71], to see how effective they must be to eliminate or stall tumor progression. Targeting cancer stem cells has been a goal of several studies, for reviews see [15, 72], and several cancer stem cell drugs have been shown to significantly decrease mammosphere formation [73]. Multiple doses of a stem cell targeted drug that is greater than 50% effective eventually kills off all the stem cells which leads to a gradual regression of the tumor. We found that killing 50% of cancer stem cells slows its progression but the tumor eventually recovers as long as a stem cell remains. Even if all but one stem cell is killed, tumor growth is only slowed for a time and then it starts growing again. Thus, while killing stem cells may seem like a therapeutic goal, these simulations suggest that suppressing a stem cell’s symmetric division rate might be a more effective or an alternative strategy. These results also suggest that using other therapeutics, such as chemotherapy, along with the cancer stem cell targeted drug should be more effective at causing a quicker tumor regression.

Hypoxia was found to decrease proliferation and increase migration of cancer cells in vitro. Hypoxic tumors grew faster, possibly due to the free space left by migratory cells. Consistent with this hypothesis, we found that tumors with slower migration rates could actually decrease the tumor growth under hypoxic conditions, probably due to the decrease in proliferation (data not shown). Thus reducing migration rate may be an effective anti-tumor therapy. While the proliferation rate under hypoxia may be decreased, the ability for stem cells to symmetrically divide may be increased due to the free space left by tumor cell migration. Hypoxia has also been shown to reduce proliferation and increase migration in in vivo tumors [74]. Hypoxia causes cells to overexpress hypoxia-inducible factor 1 alpha (HIF1α) and vascular endothelial cell growth factor (VEGF) [75] which is associated with unfavorable prognosis in breast cancer patients [76, 77]. These factors contribute to the angiogenic switch by recruiting new vasculature to the tumor [78]. Thus, while hypoxia before the angiogenic switch slows proliferation rates, the overexpression of HIF1α and VEGF and the increase in migration leads to overall increases in tumor growth. Therefore, it would be important to combine angiogenesis and tumor growth models in breast cancer in future computational models.

Hypoxia was found to increase the invasive morphology of the tumors. This is most likely due to the fact that these tumors are less proliferative and more migratory, which was found to be more finger-like in the *in silico* parameter space. Cells are able to migrate more frequently, causing fingering morphologies, but without proliferating to create a solid morphology. This leads to hypoxic cells causing a more spread-out morphology and these tumors are more likely to be invasive and possibly more metastatic. This is consistent with many studies associating in vitro/in vivo hypoxia with breast cancer invasiveness [79] and metastasis [80, 81]. On the other hand, maraviroc treatment was found to decrease the invasive morphology of the *in silico* tumors. This is due to the reduction in migration leading to fewer migrating cells and fewer fingers.

Lastly, since the morphology of the tumors is an important aspect of the invasiveness of a tumor, shape metrics have been used to classify breast tumors from mammograms [53, 54]. Therefore, to relate our simulations to clinical results, we used several shape metrics to determine whether the tumors were more invasive or more solid. Chord length, chord ratio, moment of inertia and fractal dimension were all able to predict whether the tumor was invasive or solid. Circularity and fractal dimension were used to predict benign or malignant tendencies and were statistically significant [53]. Circularity is the mean radial distance of the tumor boundary divided by the standard deviation. In breast cancer patients, circularity of malignant breast tumors were 3.62 on average, whereas benign tumors had an average value of 5.57 [53]. The fractal dimensions of the boundary of breast tumors in cross section were 1.25 for benign and 1.6 for malignant tumors [67], for brain tumors the fractal dimensions of the tumor surface were between 2 and 3 depending on the image processing method [65]. When applied to our simulated tumors, these metrics yielded circularity values between 2.6 and 4.0 and fractal dimension values between 2.3 and 2.36. According to this, all of the tumors would be predicted to be malignant, which is consistent with MB231 tumors being metastatic.

There are several limitations of this model. First of all, we examine tumor growth before the angiogenic switch. This allows us to understand the progression of early tumor but it will need to be expanded upon to understand the continued growth of the tumor, tumor vasculature, and metastasis. Another limitation of the model is that it does not take into account the complex anisotropic structure of the host mammary tissue with its branching network of mammary ducts; this background could have an effect on the shape of the growing breast tumor. This issue deserves an investigation in future models. Another limitation is that the model uses triple-negative breast cancer cell lines for parameter fittings. While this allows direct comparison between the more and less metastatic cell lines, it may not be generalizable to other types of breast cancer or solid tumors. This model focuses on primary tumor growth but in future directions we will examine breast cancer metastasis to other organs such as the lung, lymph nodes and bone.

## Conclusions

In conclusion, we have used experimental and computational techniques to examine the effects of migration of CCR5+ cancer cells, stem cell proliferation, and hypoxia on the growth and progression of triple-negative breast tumors. The major conclusions of our computational model are that 1) stem cell percentages and proliferation rates have a greater effect on tumor growth than CCR5+ percentages and migration rates, 2) treatments that do not eliminate all stem cells cause tumor reduction but the tumors eventually relapse, 3) maraviroc treatment slightly decreases tumor size, but the effect is not as pronounced as the effects of hypoxia or anti-stem cell treatment, 4) hypoxia accelerates tumor growth, 5) 3D finger-like tumor morphologies occur at higher migration and lower proliferation rates. We show that there is an important interplay between cancer stem cells, cancer CCR5+ cells, hypoxia, and therapies that can affect tumor growth and invasion. The model provides a framework for analyzing the effects of tumor cellular heterogeneity and pharmacodynamics; building on this development future studies should include other cell types in the tumor microenvironment, e.g., tumor-associated fibroblasts, and immune cells such as macrophages and T cells, as well as expand on the pharmaceutical agents that interfere with different cellular processes.

## Additional file

Additional file 1: This includes a table with the parameters used in the model, as well as the experimental measurements of CCR5 and stem cells in MB231 cells using flow cytometry, migration assays of hypoxic and normoxic MB231 cells. (DOCX 973 kb)

## Abbreviations

CCL5: Chemokine ligand 5
CCR5: Chemokine receptor 5
DCIS: Ductal carcinoma in situ
HER2: Human epidermal growth factor receptor 2
HIF1α: Hypoxia-inducible factor 1 alpha
HIV: Human immunodeficiency virus
IL6: Interleukin 6
MB321: MDA-MB-231
MR: Migration rate
PR: Proliferation rate
TNBC: Triple negative breast cancer
VEGF: Vascular endothelial growth factor
