# Supplementary material for: An agent-based model of triple-negative breast cancer: the interplay between chemokine receptor CCR5 expression, cancer stem cells, and hypoxia
Source: BMC Syst Biol. 2017 Jul 11;11:68. doi: 10.1186/s12918-017-0445-x (PMC5504656; doi:10.1186/s12918-017-0445-x)
Supplement: Additional file 1: — This includes a table with the parameters used in the model, as well as the experimental measurements of CCR5 and stem cells in MB231 cells using flow cytometry, migration assays of hypoxic and normoxic MB231 cells. (DOCX 973 kb) [file 12918_2017_445_MOESM1_ESM.docx]

**Supplemental Data**

**Methods**

Model Parameters

We show in Table 1 the list of parameters used in the agent-based model and the literature references supporting these values.

| Parameter | Value (Default) | References |
| --- | --- | --- |
| Symmetric division rate | 0.05/day | [36] |
| Stem cell division rate | 0.1-0.4/day (0.2) | [36] |
| Progenitor cell division rate | 0.5/day | [36] |
| CCR5+ migration rate | 8.33 μ/hr | [7,64-66] |
| CCR5- migration rate | 0.83 μ/hr | [62-63] |
| Senescent cell death rate | 0.1/day | [36] |
| Division limit | 12 times | [36] |
| Cell diameter | 20 μ | [[53]](file:///C:\Users\Kerri\Documents\Kerri's%20Work%202017\BMCSysBioPaperRevision\ParameterList.xlsx#RANGE!_ENREF_53) |
| CCR5+ cell percentages | 1-13% (6%) | [7, 54] |
| Stem cell percentages | 1-34% (20%) | [55-61] |
| Hypoxia migration rate | 3-fold | [67] |
| Hypoxia proliferation rate | 0.5-fold | [67] |
| CCR5+ production rate | 5% | [7, 54] |
| Hypoxic CCR5+ production rate | 25% | [53, 67] |

Cell culture

MDA-MB-231 (MB231_1) and MDA-MB-231-luc-D3H2LN (MB231_2) cells were cultured in RPMI media (Gibco; Life Technologies; Thermo Fisher Scientific, Waltham, MA, USA) with 10% fetal bovine serum (FBS, Gibco) and 1% penicillin/streptomycin (Sigma). MDA-MB-231 were gifts from Dr. Zaver Bhujwalla (JHMI, Radiology and Oncology) and MDA-MB-231-luc-D3H2LN were purchased from Caliper.

Flow cytometry

Flow cytometry was conducted using the same protocols as in our earlier papers ([53](#_ENREF_53), [54](#_ENREF_54)). Cells were washed in 10 ml of phosphate buffered saline DPBS without calcium or magnesium (Gibco, 14190) which was then removed from the flask. 3 ml of trypLE (Gibco 12604-013; Thermo Fisher Scientific, Waltham, MA, USA) was added to disassociate the cells from the flask. Once cells rounded up, 3 ml of trypsin neutralization solution (TNS) (Gibco R002100) was used to stop the reaction. Cells were counted, collected by centrifugation, and resuspended at 10^5^ cells/25 μL in stain buffer (BD Biosciences, San Jose, CA, USA). 25 μL of cells were transferred to round bottom tubes (BD Biosciences 352008) and 10 μL of CD24 antibodies labeled with allophycocyanin (APC), CD44 antibodies labeled with Peridinin chlorophyll (PerCP), or CCR5 antibodies labeled with phycoerythrin (PE)-labeled (R&D Systems) were added to the cells in the dark. The samples were incubated at 4°C for 45 minutes. The samples were washed twice with 4ml of stain buffer and collected by centrifugation. The Quantibrite PE (BD Biosciences, 340495) beads, which we use for quantification underwent the same wash and centrifugation steps. After the second wash the stain buffer was removed and 150 μL of stain buffer was added to each tube on ice. Surface receptors numbers were measured in a FACSCalibur flow cytometer (BD Biosciences).

Migration assay

50,000 MB231 cells were seeded in the top chamber of a Cell Migration plate in 0.5% serum media (CIM-plates) (ACEA Biosciences). The top compartment of the plate is separated from the bottom compartment by a microporous (8 μ) polycarbonate membrane. Media with 0.5% serum was added to the bottom compartment of the chamber and the plate was incubated at 37°C for 24 h. The sensors integrated on the bottom side of the membrane allow for immediate and real-time, continuous monitoring of cells with a real-time cell analyzer as they move and attach and spread on the gold electrodes on the underside of the top chamber. The number of cells migrated is registered as a change in cell index.

Hypoxia treatment

250,000 MB231 cells were plated into wells of a 6-well tissue culture plate and allowed to grow for 24 hours. Twenty microliters of a 15mM solution of cobalt chloride, a hypoxia inducing agent ([55](#_ENREF_55), [56](#_ENREF_56)), was added to the cells growing in 2ml of RPMI media. The cells were incubated at 4°C for 30 minutes.

**Results**

Triple-negative breast cancer cell-surface numbers for CCR5, CD44 and CD24

Previous work has shown the importance of CCR5 in the metastasis of TNBC to the lungs and lymph nodes ([5](#_ENREF_5)) and that TNBC has two populations of cells with differing numbers of CCR5 cell surface receptors ([54](#_ENREF_54)). Through modeling we have also demonstrated the importance of cancer stem cells for tumor growth ([36](#_ENREF_36)). For these reasons, we were interested in knowing the distribution of CCR5 receptors and stem cells in MB231 cell lines. There are several common markers for breast cancer stem cells, such as CD44+/CD24-, CD44+/CD24-/ESA+, ALDH1, and CD133 ([16](#_ENREF_16), [65](#_ENREF_65), [77](#_ENREF_77)). We measured the cell surface expression of CD44, CD24, and CCR5 on MB231s using quantitative flow cytometry. CCR5 was labeled using PE-conjugated antibodies, and CD44 and CD24 labeled with PerCP- and APC-conjugated antibodies respectively. We gated the CCR5 receptor high population, Supplemental Figure 1A, and the CD44 high and CD22 low receptor population, Supplemental Figure 1B, to determine the frequencies in each cell line. The frequencies of CCR5 high cells were low in both cell lines, with a mean of 6% and a standard deviation of 0.10 in MB231_2 cells and a mean of 2% and a standard deviation of 0.06 in MB231_2 cells, Supplemental Figure 1. These numbers were taken from one experiment with three replicates. In other experiments we found ranges of CC5+ cells in MB231 cell lines between 1%-8%. We found that stem cells make up 20% with (1.31 standard deviation) of the MB231_2 population with 1% being CCR5+ and they make up 9% (1.17 standard deviation) of the MB32_1 population with 1% being CCR5+, Figure 2. These numbers were the mean of three replicates.

Hypoxia increases *in vitro* cancer cell migration

Hypoxic regions are a hallmark of solid tumors, whether vascularized or not (prior to the angiogenic switch). Therefore we wanted to understand how hypoxia influences the proliferation and migration rates of MB231 cell lines *in vitro*. We treated MB231 cells with cobalt chloride for 3 hours to induce hypoxia. Cobalt chloride mimics the hypoxic response by upregulating hypoxia-inducible factor-1α (HIF-1α) and glycolytic enzymes ([56](#_ENREF_56)). We measured the migration of the hypoxic cells relative to the normal cells, Figure 3. We found that the hypoxic cells were more migratory. At 3 hours, MB231 cells were 4.4 times as migratory and at 16 hours they were 2.2 times as migratory compared to normoxic cells. Thus, for our model we assumed that hypoxic cells were 3 times as migratory as normoxic cells, which is also supported by recent experimental evidence ([71](#_ENREF_71)).


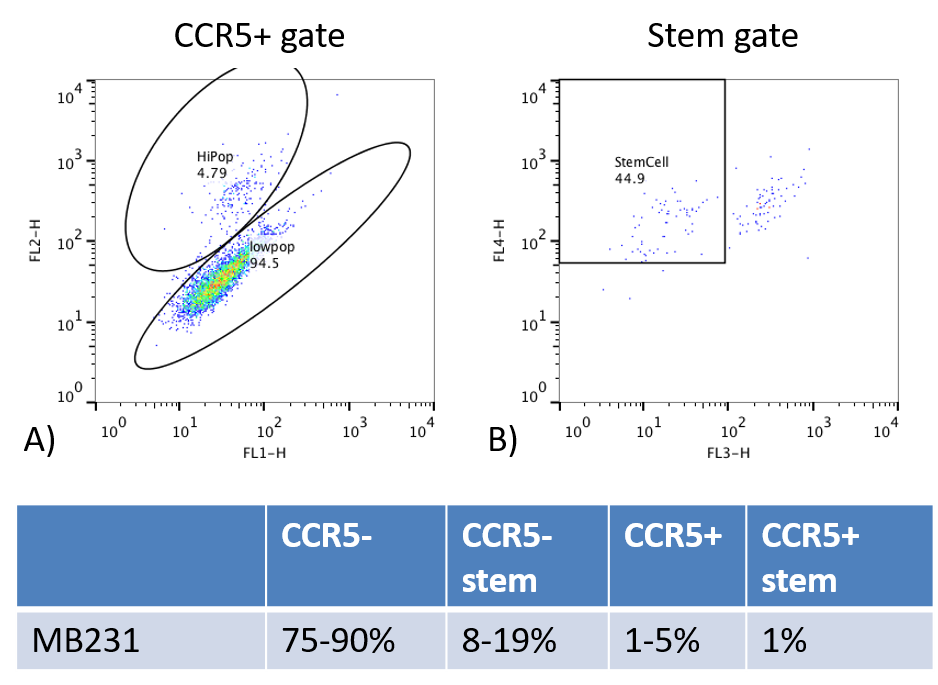


Supplemental Figure 1: Results from Flow Cytometry Analysis. We measured surface expression levels of CCR5, CD44, and CD24 in MB231 cell lines. (A) The CCR5+ population was a small subset of the overall cell population; (B) Stem cells were identified by gating CD44+ and CD24-. The percentages of each population were calculated using flow cytometry.

**
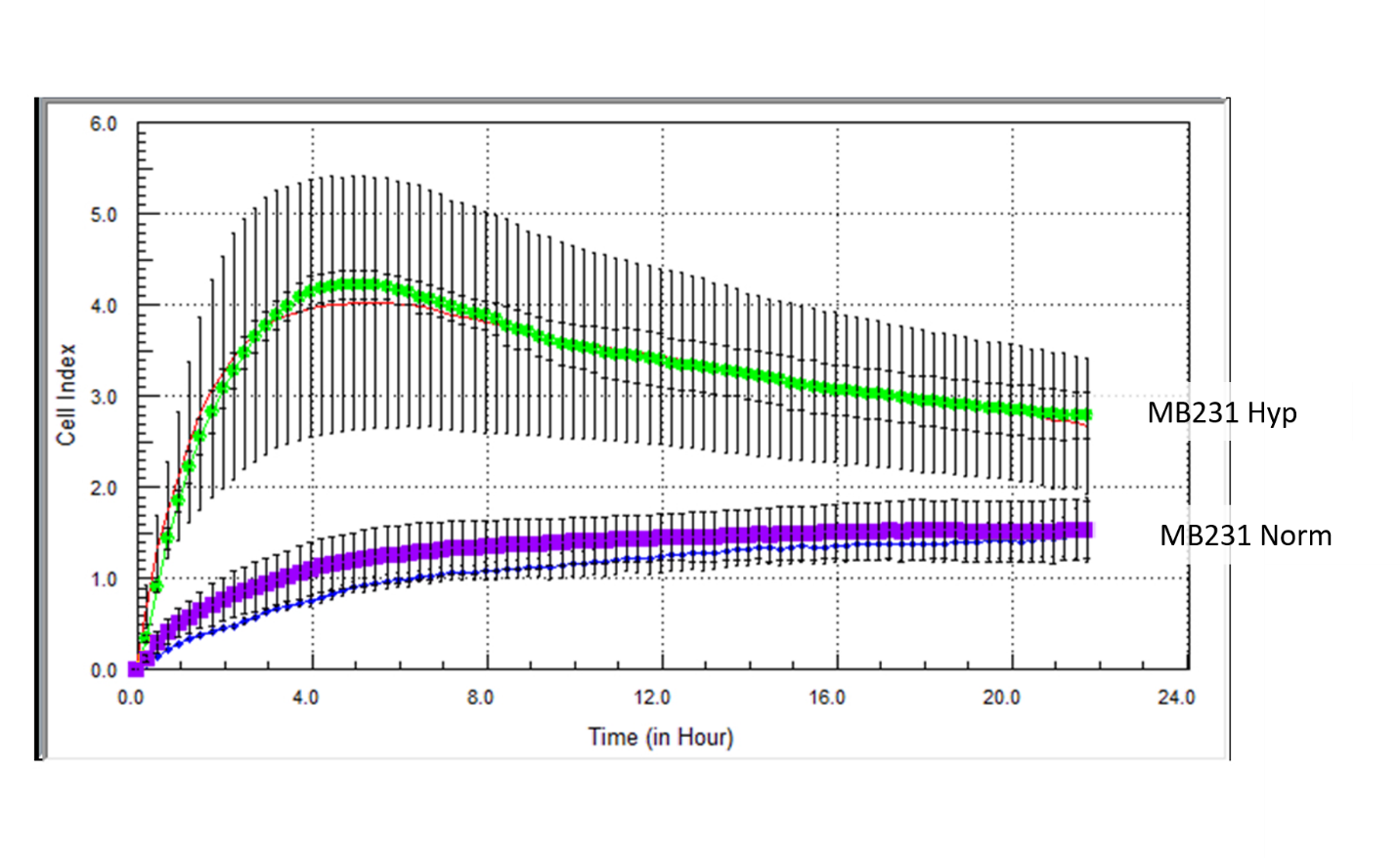
**

Supplemental Figure 2: Migration Assay of MB231 cells under Normoxic and Hypoxic Conditions. MB231 cells under hypoxic conditions are more migratory than under normoxic conditions. The error bars represent the standard deviations.
